# Application of melamine-starch and bixin film (*Bixa orellana* L.) in wound healing

**DOI:** 10.1590/acb414826

**Published:** 2026-08-14

**Authors:** Lúcia Rosa Reis de Araújo Carvalho, Maria do Amparo Veloso Magalhães, Antônio Luiz Martins Maia, Luciano Reis de Araujo Carvalho, Isabella Mousinho Marinho dos Santos, Lucielma Salmito Soares Pinto, José Figueredo Silva, Jose Zilton Lima Verde Santos, José Ribeiro dos Santos

**Affiliations:** 1 Universidade Federal do Piauí – Postgraduate Program in Biotechnology of the Northeast Biotechnology Network – Teresina (PI) – Brazil.; 2 Centro de Tecnologia Educacional em Biotecnologia – Department of Health – Teresina (PI) – Brazil.; 3 Universidade Estadual do Piauí – Department of Morphology – Center for Health Sciences – Teresina (PI) – Brazil.; 4 Universidade de São Paulo – Faculty of Dentistry – Bauru (SP) – Brazil.; 5 Hospital São Marcos – Teresina (PI) – Brazil.; 6 Universidade Estadual de Campinas – Faculty of Dentistry – Piracicaba (SP) – Brazil.; 7 Universidade Estadual do Piauí – Biotechnology Center – Teresina (PI) – Brazil.; 8 Universidade Federal do Piauí – Department of Morphology – Center for Health Sciences – Teresina (PI) – Brazil.

**Keywords:** Wound Healing, Tissues, Biotechnology, Surgical Wound

## Abstract

**Purpose:**

To evaluate through histological analysis the application of starch-melamine film and bixin (Bixa orellana L.) in wound healing in Wistar rats.

**Methods:**

Eighteen male Wistar rats were randomly assigned to three groups (n = 6 per group): Group 1 (control), cutaneous wound without topical treatment; Group 2, cutaneous wound treated with a bixin-free film; and Group 3, cutaneous wound treated with a film containing bixin. Wound healing progression was monitored by photographic documentation and histological analysis on postoperative days 3, 7, and 14.

**Results:**

At the end of three days, the three groups showed similar histological aspects with incipient re-epithelialization and edematous extracellular matrix (ECM). After seven days, the edema decreased, and the ECM appeared much more compact. After 14 days, the newly formed epidermis extended toward the wound center, but re-epithelialization was not complete in any group. Fibroblast proliferation and maturation were accentuated in all three groups. Group 3 (bixin group) exhibited wounds with a dense matrix, increased collagen deposition, and a minimal inflammatory cell infiltrate.

**Conclusion:**

The starch-melamine composite associated with bixin demonstrated potential to enhance tissue repair and modulate the inflammatory response.

## Introduction

Tissue wound healing is a complex and dynamic process supported by a series of cellular events that must be strongly coordinated to efficiently repair damaged tissue^1,2^. Factors such as wound depth, presence of infection, nutritional status, and systemic conditions of the patient can significantly influence the efficiency and quality of healing^3^.

The development of biomaterials represents a significant advance in the wound healing process, especially in chronic and difficult-to-heal wounds. These materials, such as hydrogels, biodegradable films, scaffolds, and bioactive dressings, are designed to interact directly with the injured tissue, promoting an environment conducive to tissue regeneration. They can act by controlling moisture, releasing drugs in a localized manner, preventing infections, and stimulating cell proliferation and angiogenesis. Furthermore, biomaterials based on natural or synthetic polymers can contribute growth factors, nanoparticles, or antimicrobial compounds, which enhances their clinical efficacy^4,5^.

In this context, starch, a biodegradable natural polysaccharide, can be used as a bioadhesive due to the formation of a gel from amylose and amylopectin, glucose polymers that constitute starch^6,7^. The natural pigment from annatto seeds (*Bixa orellana* L.), also called bixin, can be added to the starch-based adhesive, providing additional bioactive properties, since annatto is commonly used as an anti-hemorrhagic, expectorant in syrup form, for gargling, as a laxative, stomachic, bioactive compound, and against dyspepsia, in addition to having antimicrobial potential^8^.

To prevent the glue from becoming thin and brittle, it is necessary to incorporate an agent that promotes waterproofing and plasticization of the material^2^. One substance that can act in this context is melamine, which is weakly alkaline and used in the manufacture of plastics along with formaldehyde^9,10^.

Thus, this study aimed to investigate, through Wistar rats, the potential application in wound healing of starch-melamine composite with bixin in cutaneous wounds.

## Methods

This study was approved by the Animal Research Ethics Committee of the Universidade Estadual do Piauí (UESPI), under protocol number 033343/2024-11, in accordance with the Guiding Principles for the Care and Use of Laboratory Animals. The study was conducted using 18 adult-male Wistar rats (*Rattus norvegicus*), 90 days old, with an average body weight between 250 and 260 grams, obtained from the UESPI’s animal facility. The rats were kept in standardized polypropylene cages, with six animals each, under controlled conditions of temperature (25°C), ambient humidity, and 12/12 hour light/dark cycles. They had unrestricted access to water and standard food as needed.

The following reagents were used as raw materials for the research: soluble starch P.A. (ACS, Dinâmica Química Contemporânea LTDA, LOT 106850); melamine 99% (Chemistry Aldrich); formaldehyde PA ACS (Formalin); glycerin PA ACS; bixin; and KOH PA ACS. All reagents and analyses were provided and performed by the Bio-Electrochemical Research Laboratory of the Chemistry Department at Universidade Federal do Piauí.

### Starch-melamine and bixin film

To obtain the composite, 1 g of melamine and 1 g of soluble starch were used. To the melamine, 1.5 mL of formaldehyde, 20 mL of water heated to 60°C, and KOH were added until the pH was adjusted to 11. The solution was then heated and stirred at 60°C for 30 minutes up to complete dissolution. Simultaneously, the starch was slowly dissolved in 20 mL of water at 60°C with the addition of glycerin. The solutions were then mixed and kept under controlled heating at 85°C for 30 minutes, initially forming a transparent gel with an average viscosity of 865.2 mPa. The material was subsequently distributed into Petri dishes and subjected to drying in an oven between 40 and 45°C until a white, homogeneous, and hygroscopic polymeric film was obtained, produced in duplicate. After drying, 5 mg of bixin were incorporated onto the surface of the film, obtaining the final composite intended for application to experimental skin lesions^11^.

The structural characterization of the film was performed by Fourier transform infrared spectroscopy (FTIR), Raman spectroscopy, X-ray diffraction (XRD), and ultraviolet-visible (UV-Vis) spectroscopy. The FTIR spectra demonstrated the presence of the main functional groups’ characteristics—starch, melamine, and bixin —, without significant structural changes after the formation of the composite. The results suggested a predominance of intermolecular physical interactions, especially hydrogen bonds between the hydroxyl groups of starch and bixin and the amine groups of melamine, indicating adequate miscibility between the components of the polymeric matrix^11^.

Raman spectroscopy analysis revealed characteristic bands of starch and melamine, as well as spectral changes after the incorporation of bixin, mainly in the region between 977 and 997 cm^-1^, suggesting molecular interaction between the film constituents. Low-intensity bands related to the formation of chemical interactions in the ternary composition were also observed, indicating the formation of a homogeneous composite potentially applicable in tissue repair processes^11^.

Evaluation in the UV-Vis region demonstrated the ability of bixin incorporated into the film to release its contents. Spectrophotometric analysis revealed the presence of characteristic bixin absorptions in the 455-nm region, observed mainly after approximately 10 minutes of immersion of the film in an aqueous medium, indicating gradual release of the bioactive compound from the polymeric matrix^11^.

The preliminary biocompatibility of the material was investigated using the *Allium cepa* bioassay. The results demonstrated no significant toxicity, as no important reductions in root growth, alterations in the mitotic index, micronucleus formation, or chromosomal aberrations were observed when compared to the negative control. These findings suggest an absence of relevant cytotoxic, mutagenic, and genotoxic effects of the bixin film under the evaluated conditions^11^.

### Induction of experimental injury

The 18 animals were randomly divided into three groups (n = 6 in each group):

- Group 1 (control): cutaneous wound without topical treatment;
- Group 2: cutaneous wound treated with a bixin-free film;
- Group 3: cutaneous wound treated with a film containing bixin.

**Figure 1:**
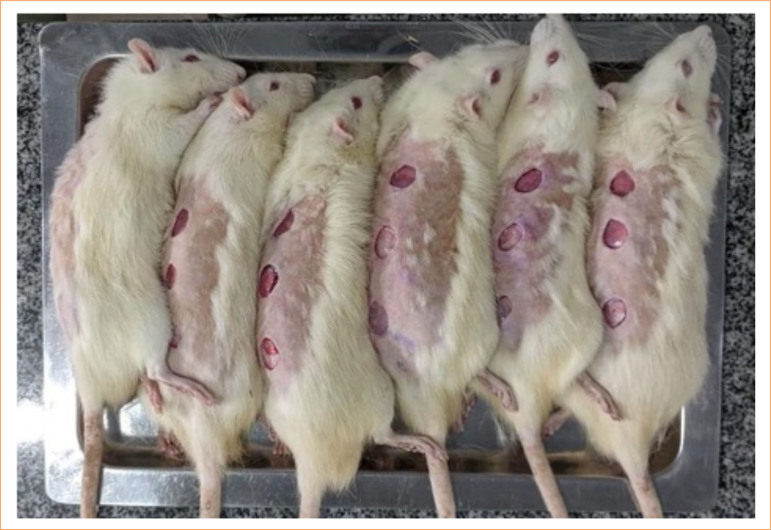
Dorsal view of the animals after trichotomy and induction of the surgical wound.

Source: Elaborated by the authors.

Dissociative anesthesia, composed of xylazine and ketamine, was used in a 1:1 ratio, administering 0.1 mL for every 100 g of body weight. Therefore, each animal received 0.03 mL of the anesthesia intramuscularly. Then, trichotomy was performed on the dorsal part followed by induction of the surgical wound. For this, a 2-cm diameter metal punch was used to mark a circle on the dorsal skin of each rat. Under anesthesia, the incision was made with the aid of a 15-cm curved/blunt surgical scissors. Therefore, the marked skin was completely removed, deepening the incision until the dorsal muscle fascia was visualized, culminating in the creation of the standard experimental wound (Fig. 1).

**Figure 2:**
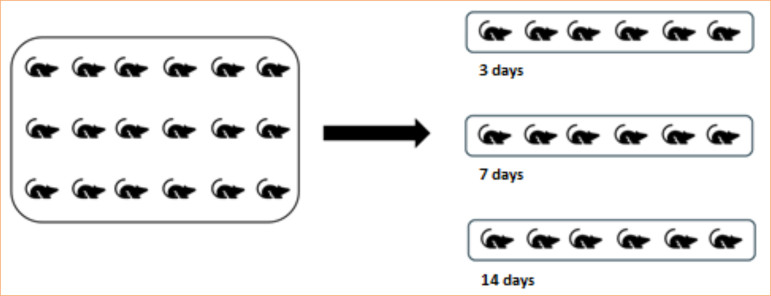
Schematic representation of the distribution of the groups studied.

Source: Elaborated by the authors.

The animals in groups 2 and 3, after the surgical procedure, were immediately covered with the appropriate polymeric film (without and with bixin, respectively), directly on the lesion area. The animals in the control group did not receive any type of local therapy, serving as a control group. The distribution of animals for the various experimental periods was done according to the scheme shown in Fig. 2.

**Figure 3:**
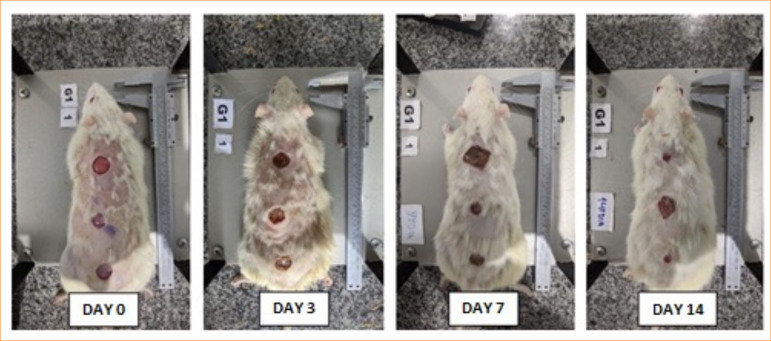
Macroscopic analysis of the injured area. The wound area was evaluated on each day of the experiment in rats 1, 2, 3, 4, 5, and 6 of each group, respectively.

Source: Elaborated by the authors.

The wound healing progression was monitored through photographic records for macroscopic evaluation of the injured area on postoperative days 3, 7, and 14 (Fig. 3). During the same period, tissue samples were collected from each animal in each group for microscopic analysis. The samples, encompassing the entire area of the lesion and adjacent tissue margins, were carefully removed in an elliptical shape using surgical scissors and placed in containers with a 10% buffered formaldehyde solution. Euthanasia was performed at the end of the 14th day by cervical dislocation, necessarily preceded by deep anesthesia with a combination of xylazine and ketamine.

### Histological analysis

The tissue samples underwent routine histological processing and were embedded in paraffin. Subsequently, the paraffin blocks were sectioned using a Lupetec MRP 09 microtome (São Carlos, SP, Brazil), obtaining sections 5-μm thick. For the qualitative histological study and semi-quantitative analysis, the histological sections were stained with hematoxylin-eosin (HE) and examined using an Olympus CX31 trinocular optical microscope, model YS100, equipped with a digital camera (Bell & Howell, EU 16.0 Plus, United States of America). For comparison of the groups in the qualitative analysis, the histological preparations were photographed in the center of the lesions.

The qualitative histological analysis of the inflammatory reaction was based on the evaluation of re-epithelialization, granulation tissue, inflammatory cell infiltrate, fibroblasts, collagen deposition, and neovascularization. The criteria for the histological analysis followed the criteria of the protocol described by Meirelles et al.^10^.

## Results and discussion

According to Carvalho et al.^11^, the composite formation occurred in two stages: the preparation of methylol-melamine and starch gel. The two parts were mixed under stirring and heating until a gel was formed. A viscosity test was performed to characterize the gel. The film was obtained by drying it in an oven at 40°C in a Petri dish. Bixin was added to the film using a bixin solution. The film was characterized by FTIR, Raman, XRD, UV-Vis, moisture, toxicity testing, and bixin release techniques. The gel viscosity was similar to that of starch gel. Drying the gel resulted in an amorphous, white, hygroscopic film. After adding bixin, the film color changed to yellow or reddish. The roots of *A. cepa* were not altered. No death was observed in the *Artemia salina* test. The bixin release test indicated release after 10 minutes. The obtained gel was dried at low temperatures to form a film. The film characterization indicated the presence of the initial components and presented a homogeneous phase. Bixin was released from the film, which supports its potential application in wound treatment^11^.

**Figure 4:**
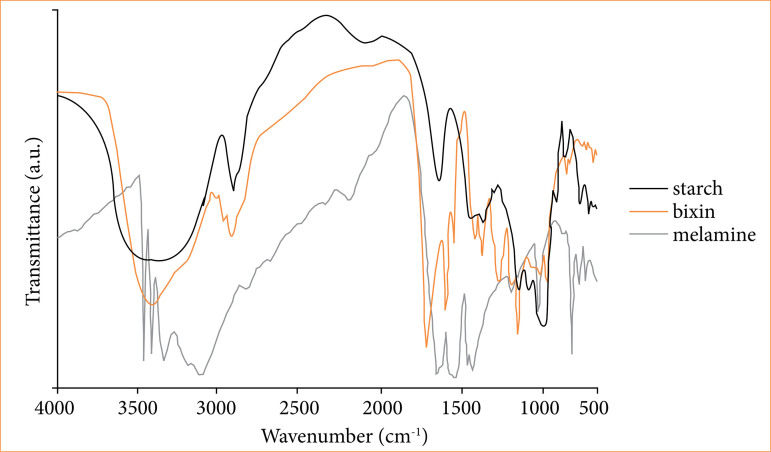
FTIR of starch, melamine and bixin.

Source: Elaborated by the authors.

Figure 4 shows the infrared spectrum for soluble PA starch. The characteristic peak at 3,422 cm^-1^ is generated by O–H stretching vibration, while the band at 2,930 cm^-1^ was associated with CH_2_ stretching. At 1,650 cm^-1^, the observed band is due to CO carbonyl stretching, and the region from 1,152 to 1,005 cm-1 applies to the fingerprint area for polysaccharides^12^.

In the infrared spectrum of melamine (Fig. 4), it is possible to observe the NH stretching in the region of 3,000 to 3,500 cm^-1^, with bands at 1,646 and 1,432 cm^-1^ corresponding to the bending of the amino groups. At 3,328 cm^-1^, the sharp peak is related to the asymmetric stretching of the -NH_2_ group and its symmetric stretching at 3,135 cm^-1^. At wavenumber 1,554 cm^-1^, the stretching related to the 1,3,4-s-triazine ring occurred, at 1,436 cm^-1^, related to the CN group of the side chain, and at 1,020 cm^-1^, the CN stretching of primary amines linked to the primary carbon. The band at 810 cm-1 is characteristic of melamine, relating to out-of-plane vibrations of ring^13^.

**Figure 5:**
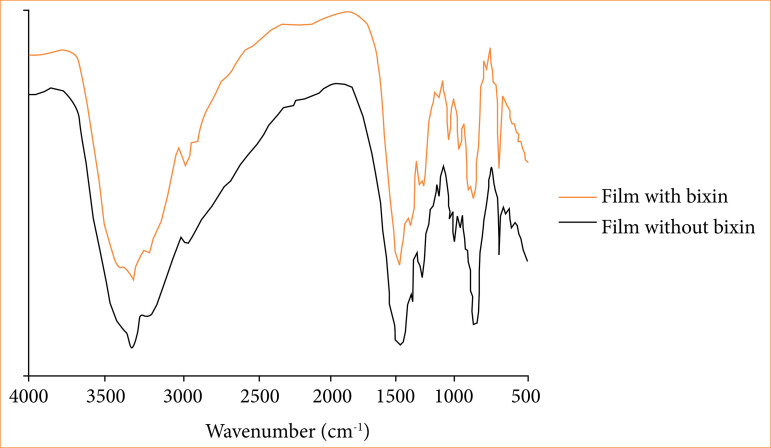
Fourier transform infrared spectroscopy of films with and without bixin.

Source: Elaborated by the authors.

The FTIR spectrum of bixin (Fig. 5) showed a characteristic band of the hydroxyl group at 3,415 cm^-1^, and at 1,718 cm^-1^ an α,β-unsaturated carbonyl ester was seen. The spectrum also showed stretching bands for C=C at 1,606 and 1,561 cm^-1^. CH_4_ *sp vibrations* ^3^ (2,955 cm^-1^), CH *sp* ^2^ (3,034 cm^-1^) and CO of the ester (1,163 cm^-1^) were also observed^14^.

**Figure 6:**
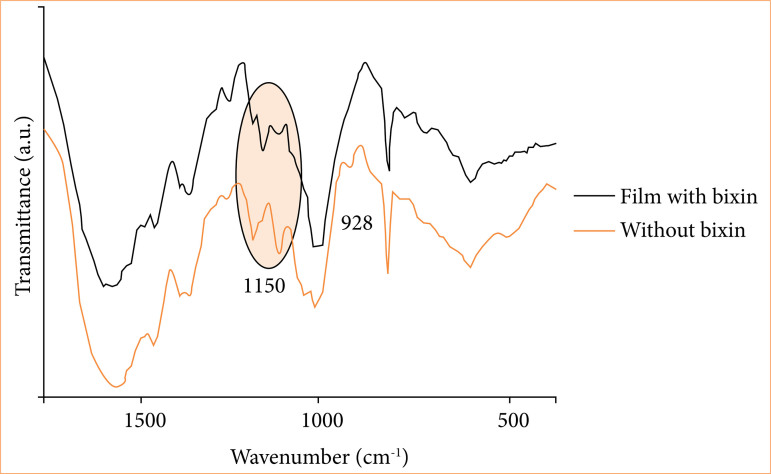
Fourier transform infrared spectroscopy from 1,400 to 400 cm^-1^ of the films with and without bixin.

Source: Elaborated by the authors.

The peaks for the film with bixin were similar to those of the spectra of the film without bixin (Fig. 6). The locations of the peaks of the functional groups in the spectra of the films did not change significantly in relation to their precursors, indicating that the ternary composition cannot alter the structure of the components, with mainly physical interactions promoting changes in peak intensities^14^. However, it is possible to observe a difference in the spectra in the 1,150-cm^-1^ range, suggesting that bixin is binding through -H (hydrogen) bonds with starch^15^.

The band at 928 cm^-1^ related to glycosidic bonds, presented at 934 cm-1 for starch, shifted to a shorter length. It may suggest an increase in electron density around the COC bond of starch, with hydrogen interactions with the -NH_2_ groups of melamine and -OH of bixin, causing a reduction in bond length. Therefore, it can be stated that there is miscibility in the ternary composition of the films^16^.

**Figure 7:**
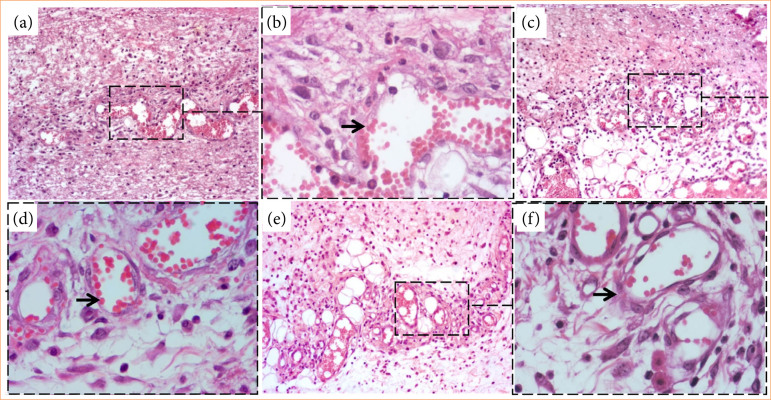
Histological aspects after three days of evolution. (a and b) Group 1 (control), (c and d) group 2 and (e and f) group 3 show wounds covered by a fibrinoneutrophilic crust. The wound bed is occupied by highly edematous extracellular matrix, containing numerous venules and congested blood capillaries (arrows). There is intense inflammatory cell infiltrate composed predominantly of neutrophils. Hematoxylin and eosin (a, c and e) 100x; (b, d and f) 400x.

Source: Elaborated by the authors.

Regarding the analysis of the tissue wounds, after the end of the three-day follow-up period, it was observed that the three groups showed similar histological aspects. Re-epithelialization was incipient, limited to the periphery and still very distant from the central area of the wounds, which were covered by a crust of variable thickness, formed by fibrinoneutrophilic material. The wound bed was occupied by young granulation tissue, characterized by a highly edematous extracellular matrix (ECM), containing numerous congested venules and blood capillaries, as well as intense inflammatory cell infiltrate composed predominantly of neutrophils, in addition to macrophages (Fig. 7).

The histological findings observed three days after healing are consistent with the initial inflammatory phase of wound healing, characterized by the recruitment of neutrophils and macrophages to remove cellular debris and microorganisms, as well as the initial formation of granulation tissue. Despite the similarity between the groups during this initial period, the absence of exacerbated signs of necrosis or intense inflammatory reaction in the treated groups suggests adequate biocompatibility of the starch-melamine composite with bixin, corroborating previous results obtained in bioassays with *A. cepa* and *A. salina*. Furthermore, the gradual release of bixin observed in the characterization study may have contributed to maintaining a microenvironment conducive to tissue repair, since carotenoids have recognized antioxidant and anti-inflammatory activity, capable of modulating the oxidative stress present in the initial phase of wound healing^11,17,18^.

**Figure 8:**
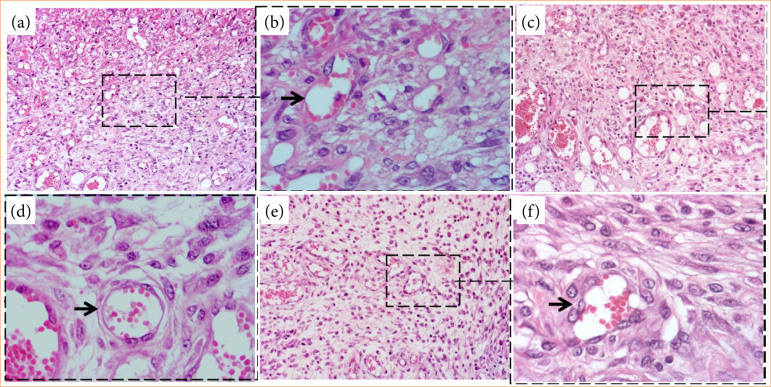
Histological aspects after seven days of evolution. In all groups, edema decreased, and neoformation of blood capillaries occurred (arrows). (a and b) In group 1 (control), intense infiltration of macrophages and neutrophils is observed. (c and d) Group 2 exhibits proliferation of randomly arranged young fibroblasts. (e and f) In group 3, the fibroblasts are apparently more numerous and arranged in bundles parallel to the skin surface. Total re-epithelialization of the wounds did not occur in any of the groups. Hematoxylin and eosin (a, c and e) 100x; (b, d and f) 400x.

Source: Elaborated by the authors.

After seven days of evolution, the edema was substantially reduced, and the ECM appeared much more compact than at three days of evolution, containing young fibroblasts and newly formed blood capillaries. The control group still exhibited intense infiltration of macrophages and neutrophils. In group 2, the young fibroblasts were arranged in bundles with random orientation. In group 3, the fibroblasts were apparently more numerous and arranged in bundles parallel to the skin surface. Complete re-epithelialization of the wounds did not occur in any of the groups (Fig. 8).

This pattern suggests an acceleration of the proliferative process and greater organization of granulation tissue, fundamental aspects for the orderly deposition of collagen and subsequent wound remodeling. Studies demonstrate that starch-based biomaterials have a high capacity for maintaining local moisture and supporting cell migration, favoring angiogenesis and fibroblast proliferation. Furthermore, the presence of bixin may have potentiated these effects due to its antioxidant and healing properties described in the literature, reducing the prolonged persistence of the inflammatory cell infiltrate. The parallel arrangement of fibroblasts observed in the treated group also suggests greater tissue maturation when compared to the control groups, indicating that the composite may act as a temporary scaffold conducive to cell reorganization during skin repair^6,8^.

**Figure 9:**
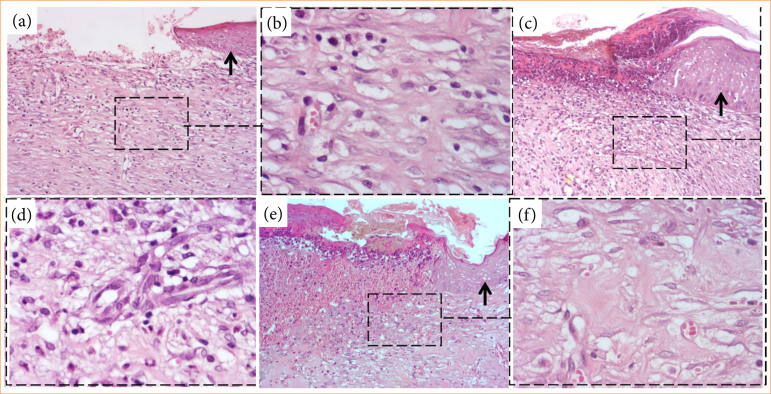
Histological aspects after 14 days of evolution. The newly formed epidermis (arrows) approached the center of the lesions, but in none of the groups, re-epithelialization was complete. The proliferation and maturation of fibroblasts increased in all three groups. In (a and b) group 1 (control) and in (c and d) group 2, there is still intense infiltration of macrophages and neutrophils in the matrix. (e and f) Group 3 exhibits wounds with a dense matrix, with increased collagen deposition and few inflammatory cells. Hematoxylin and eosin (a, c and e) 100x; (b, d and f) 400x.

Source: Elaborated by the authors.

After 14 days of evolution, the newly formed epidermis extended toward the wound center, but in none of the groups was re-epithelialization complete. The proliferation and maturation of fibroblasts were accentuated in all three groups. Group 3 exhibited wounds with a dense matrix, with increased collagen deposition and minimal inflammatory cell infiltrate (Fig. 9). These findings indicate that the composite containing bixin favored a more efficient progression from the proliferative phase to the tissue remodeling phase. The increased collagen deposition suggests increased fibroblastic activity and better matrix organization, factors directly related to the gain in mechanical resistance of the wound^11,17^.

Although re-epithelialization was not complete in any group, the histological profile found in the treated group shows a more advanced scarring process. Previous studies demonstrate that polymeric biomaterials associated with natural bioactive compounds can reduce inflammatory time and stimulate collagen synthesis through the modulation of inflammatory cytokines and oxidative stress^11,17^. Thus, the results found suggest that the starch-melamine composite incorporated with bixin presents promising potential as a biomaterial for application in cutaneous tissue repair.

## Conclusion

The starch-melamine composite combined with bixin demonstrated potential to enhance tissue repair and modulate the inflammatory response. Further studies are needed to fully evaluate the potential of this composite.

## Data Availability

All data sets were generated or analyzed in the current study.
